# Taxonomic hypotheses regarding the genus *Gerbillus* (Rodentia, Muridae, Gerbillinae) based on molecular analyses of museum specimens

**DOI:** 10.3897/zookeys.566.7317

**Published:** 2016-02-18

**Authors:** Arame Ndiaye, Caroline Tatard, William Stanley, Laurent Granjon

**Affiliations:** 1 Département de Biologie Animale, Faculté des Sciences et Techniques, Université Cheikh Anta Diop, Dakar, BP 5005, Senegal; 2 BIOPASS (IRD-CBGP/ISRA/UCAD), Campus de Bel-Air, BP 1386, CP 18524 Dakar, Senegal; 3 UMR CBGP (INRA⁄IRD⁄CIRAD⁄Montpellier SupAgro), Campus International de Baillarguet, CS 30016, 34988 Montferrier-sur-Lez cedex, France; 4 Science and Education, Field Museum of Natural History, 1400 South Lake Shore Drive, Chicago, Illinois 60605, U.S.A.

**Keywords:** Cytochrome b, degraded DNA, synonymy, systematics, vicariance

## Abstract

Methodological improvements now allow routine analyses of highly degraded DNA samples as found in museum specimens. Using these methods could be useful in studying such groups as rodents of the genus *Gerbillus* for which i) the taxonomy is still highly debated, ii) collection of fresh specimens may prove difficult. Here we address precise taxonomic questions using a small portion of the cytochrome *b* gene obtained from 45 dry skin/skull museum samples (from 1913 to 1974) originating from two African and three Asian countries. The specimens were labelled *Gerbillus gerbillus*, *Gerbillus andersoni*, *Gerbillus nanus*, *Gerbillus amoenus*, *Gerbillus perpallidus* and *Gerbillus pyramidum*, and molecular results mostly confirmed these assignations. The close relationship between *Gerbillus nanus* (Asian origin) and *Gerbillus amoenus* (African origin) confirmed that they represent vicariant sibling species which differentiated in allopatry on either side of the Red Sea. In the closely related *Gerbillus perpallidus* and *Gerbillus pyramidum*, specimens considered as belonging to one *Gerbillus pyramidum* subspecies (*Gerbillus pyramidum floweri*) appeared closer to *Gerbillus perpallidus* suggesting that they (*Gerbillus pyramidum floweri* and *Gerbillus perpallidus*) may represent a unique species, distributed on both sides of the Nile River, for which the correct name should be *Gerbillus floweri*. Furthermore, the three other *Gerbillus pyramidum* subspecies grouped together with no apparent genetic structure suggesting that they may not yet represent genetically differentiated lineages. This study confirms the importance of using these methods on museum samples, which can open new perspectives in this particular group as well as in other groups of interest.

## Introduction

DNA sequences have proven useful in taxonomic studies, and they now represent a primary source of information when it comes to the delimitation of species (Wiens 2007). Used in combination with other sources of data in the frame of integrative taxonomy (Dayrat 2005), they often provide convincing arguments for, or against the recognition of taxa as distinct species. DNA is especially useful in the case of cryptic species, where morphological criteria fail to unambiguously identify specific taxa (Knowlton 1986). For more than 25 years now, the recovery of DNA from ancient paleontological, archaeological and historic study specimens is routinely conducted (Pääbo 1989, Cooper 1994). The analysis of such DNA sequences has been instrumental in clarifying the systematics of extinct taxa, but it can also be of help in modern taxa that are difficult to sample today. This may happen when they are endangered or vulnerable in the wild, but also when their distribution interferes with sensible human activities and / or is situated in areas of conflict. In these cases, the use of museum specimens dating from periods and coming from areas where collection activities were easier could represent a convenient way of getting molecular information from particular species / populations. However, there are some limits to using such materials. The primary concern is that the sequences obtained are usually of relatively small size, due to the degraded state of the DNA of museum specimens. Given this limitation, the choice of the gene that will be targeted is of special concern: it has to be sufficiently variable to contain enough information, even in a small fragment, to allow distinguishing a particular species from its sister and other closely allied ones. Sequences as short as one hundred base pairs long have been shown to meet these requirements, for genes like cytochrome oxdydase 1 (CO1) in insects, or cytochrome *b*
(cytb) in rodents (Hajibabaei et al. 2006, Galan et al. 2012).

Gerbils of the genus *Gerbillus* represent a good example where such an approach can be expected to bring significant information. The systematics of this genus, as well as the one of the Gerbillinae subfamily to which it belongs, is still intensely debated, at various taxonomic levels (Chevret and Dobigny 2005, Abiadh et al. 2010, Alhajeri et al. 2015, Ndiaye et al. in review). At the specific level, many species still await confirmation of their taxonomic status, being based on very few specimens coming from localized areas (e.g. *Gerbillus agag*, *Gerbillus burtoni*, *Gerbillus grobbeni*, *Gerbillus jamesi*, *Gerbillus muriculus*, *Gerbillus principulus*, *Gerbillus syticus*, *Gerbillus vivax*, see Musser and Carleton 2005, Granjon 2013). A number of these areas are currently difficult to access due to political instability and regional insecurity, especially in the Saharo–Sahelian area where the majority of the *Gerbillus* diversity occurs (Brito et al. 2014). Conversely, some of these areas have been sampled quite extensively for rodents in the second half of the 20^th^ century, and important collections have been gathered during this period. Examples include areas such as Sudan (Setzer 1956), Libya (Ranck 1968), and Egypt (Osborn and Helmy 1980). The last-named country is of special interest to *Gerbillus* evolutionary history. First, it is located at the junction of Africa and Asia, the two continents over which the genus *Gerbillus* is distributed. Second, it is crossed by the Nile River that may represent a significant barrier to rodent, and especially gerbil, dispersal, thus promoting potential differentiation between species or subspecies. As the collection built by Osborn and Helmy (1980) proved to be especially rich in gerbilline rodent specimens, we tried to address the following questions on the basis of partial cytochrome *b* sequences obtained from a selected sample of *Gerbillus* museum specimens:

- Is the differentiation between *Gerbillus amoenus* (from Africa) and *Gerbillus nanus* (from Asia), recently evidenced by Ndiaye et al. (2013) based on complete sequences of cyt*b*, found when using shorter sequences? If it is, then do the Egyptian specimens belong to *Gerbillus amoenus*, as should be the case? This question was addressed using museum specimens from Egypt, Pakistan and Afghanistan.
- What are the evolutionary relationships between various purported *Gerbillus pyramidum* subspecies and other *Gerbillus* species, such as *Gerbillus perpallidus*, *Gerbillus andersoni* and *Gerbillus gerbillus*?
- Do the subspecies of *Gerbillus pyramidum* listed by Osborn and Helmy (1980), based on morphological attributes (*Gerbillus pyramidum elbaensis*, *Gerbillus pyramidum floweri*, *Gerbillus pyramidum gedeedus* and *Gerbillus pyramidum pyramidum*), correspond to unique genetic clusters?

## Material and methods

Forty-five tissue samples were obtained from dry fragments that were still present on the skulls and skins of *Gerbillus* specimens from Egypt and Asia, that are housed at the Field Museum of Natural History, Chicago, USA (Suppl. material 1). These samples represent six species and were labeled as: *Gerbillus amoenus amoenus* (N = 6), *Gerbillus andersoni andersoni* (N = 5), *Gerbillus gerbillus gerbillus* (N = 4), *Gerbillus nanus* (N = 6), *Gerbillus perpallidus* (N = 5) and *Gerbillus pyramidum* (N = 19). The latter was represented in our sample by the subspecies *Gerbillus pyramidum elbaensis* (N = 5), *Gerbillus pyramidum floweri* (N = 4), *Gerbillus pyramidum gedeedus* (N = 5) and *Gerbillus pyramidum pyramidum* (N = 5). These specimens were collected from 1913 to 1974 in Egypt (N = 38), Sudan (N = 1), Afghanistan, India and Pakistan (N = 2 for each; Suppl. material 1).

DNA was extracted in the Labex CeMEB degraded DNA platform (Montpellier, France) using the QiaAmp DNA micro kit (Qiagen). Due to the degradation of DNA in museum samples, we amplified a short fragment of cyt*b* by designing two new primers named GERBCYTB-F2 (5’- GCA AAC GGA GCC TCA ATA TT - 3’) and GERBCYTB-R3 (5’-CAT TCT ACR ATT GTT GGG CCA - 3’). These primers are respectively located at positions 250 and 488 of the cyt*b* gene, delimiting a 239 base pair (bp) fragment. The 25μl reaction solution was prepared by mixing 14.5μl of DNase-RNase free water (Qiagen), 2.5μl of buffer (1X concentration), 2μl MgCl2 (2mM), 2.5μl dNTP (250μM; Sigma), 0.5μl of each primer (0.5μM), 0.5μl of AmpliTaq Gold (2.5 units; Applied biosystems). 1μl and 2μl of DNA aliquots of the extracted samples were amplified separately, and used for further comparisons. The cyt*b* amplification was done at the CBGP molecular biology platform (Montferrier-sur-Lez, France) using PCR programs on a Master Cycle rep Gradient (eppendorf), including an activation step of 95 °C for 10 min followed by 55 cycles comprising a first denaturation at 94 °C for 30 s, hybridization at 50 °C for 30 s and elongation at 72 °C for 45s. The last step was a final extension at 72 °C for 7min. Three negative controls were used to check for contamination during DNA extraction, preparation of the mix and DNA distribution. In the first control (extraction control), no tissue was added to the tube; the second control (PCR mix control) was a closed tube, with only the PCR mix; the last control (DNA distribution control) was a tube with only the PCR mix, which was open during the entire process of DNA distribution, in order to check for the presence of DNA in the air. We verified the size and quality of each amplified DNA sequence fragment by performing an electrophoretic migration on a 2% agarose gel. The PCR products obtained at both DNA concentrations were sent to Eurofins MWG (Germany) for sequencing, and the results were compared among individuals to ensure that we obtained the same amplified sequence fragment.

The sequences were then checked, aligned and edited with BioEdit v.7.1.3.0 (Hall 1999) and we added 40 sequences downloaded from GenBank (www.ncbi.nlm.nih.gov/genbank) of various well-characterized *Gerbillus* species as a reference, and *Sekeetamys calurus* was used as an outgroup (see Suppl. material 1 for details). Phylogenetic reconstructions were carried out via Neighbor-Joining (NJ) and Bayesian inference (BI) using SeaView v.4.2.12 (Gouy et al. 2010) and MrBayes v.3.1.2 (Ronquist et al. 2012), with bootstrap values (BP) and posterior probabilities (PP) used as node support in respective analyses. The best fit models for Neighbor-Joining and Bayesian reconstructions were K2P and GTR+I+G, respectively. We tested it using jModeltest v2.1.4 (Darriba et al. 2012), with default settings (11 number of substitution schemes corresponding to 88 models to test, base frequencies and rate variation with 4 categories, ML optimized for the likelihood calculations). In the latter, two independently Markov chain Monte Carlo (MCMC) runs were carried out for one million generations each. Trees were sampled every 100 generations and convergence was reached when the average standard deviation of split frequencies remained under 0.01, thus reflecting the fact that the two tree samples become increasingly similar. Finally we applied a 25% burn-in. Pairwise Kimura 2-Parameter genetic distances were obtained for our cyt*b* dataset under MEGA v6 (Tamura et al. 2013) with an associated standard error estimated based on 1000 bootstrap replicates. All codon positions were kept for analyses and no positions containing gaps and / or missing data were observed.

## Results

No contamination was recorded during this series of experiments, as testified by examination of the content of the control tubes. DNA could not be amplified from only one of the samples. Additionally, a comparison of the results obtained using amplified PCR products from two DNA concentrations showed that the obtained sequences were identical in all but seven individuals, making us suspect the presence of nuclear copies of mitochondrial DNA (*Numt*). Unambiguous sequences (239 bp) of 37 individuals were obtained, to which we added the sequences of 40 “reference” individuals of different, well-characterized, *Gerbillus* species taken from GenBank and a representative of the outgroup *Sekeetamys calurus*, leading to a final cyt*b* dataset comprising 77 individuals.

**Figure 1.**
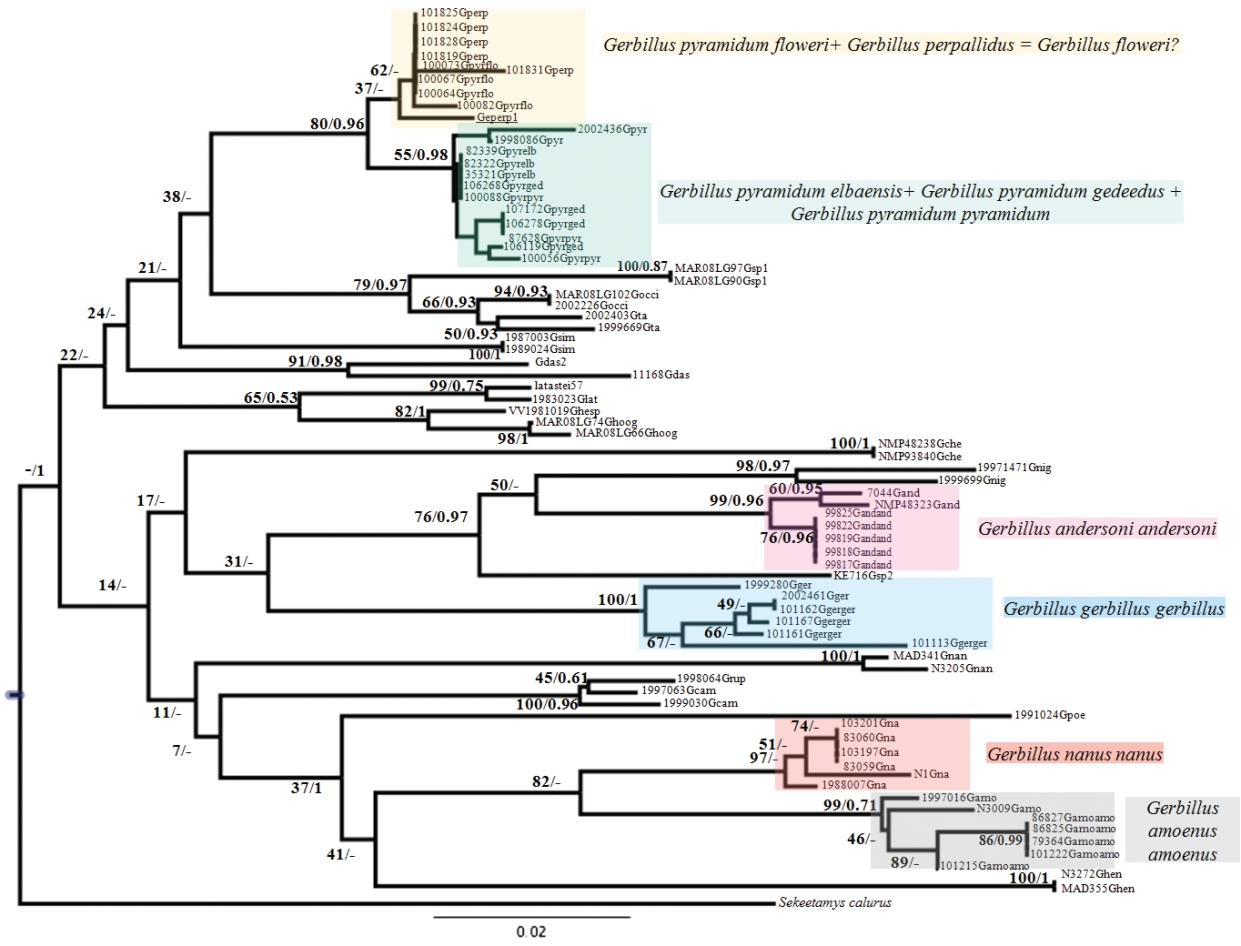
Phylogenetic reconstruction based on 239bp sequences of the cytochrome *b* gene using Neighbor-Joining. Values on nodes correspond to bootstraps / posterior probabilities respectively while “-” refer to places where both methods of reconstruction did not agree. Colored clades include museum specimens for which original sequences were obtained for the purpose of this study.

The phylogenetic tree (Fig. 1) obtained using NJ (with the bootstrap / posterior probability values indicated on nodes, respectively) presented here shows a similar topology to the BI tree with our museum specimens distributed in four main clades. The first two are unambiguously identified as *Gerbillus andersoni* (98/0.97) and *Gerbillus gerbillus* (99/1). The other two correspond to a *Gerbillus nanus* / *Gerbillus amoenus* (82/-), and a *Gerbillus perpallidus* / *Gerbillus pyramidum* (80/0.96) clade, respectively, each of which appears structured in two sub-clades. In the former, specimens referable to *Gerbillus nanus* from Asia (Pakistan and Afghanistan) cluster together (97/-) as a sister group to specimens referable to *Gerbillus amoenus* from Africa (Egypt, Niger, Mauritania; 99/0.71). The latter is divided into two sub-clades (separated by a K2P genetic distance = 0.017, see Table 1), one with *Gerbillus perpallidus* and *Gerbillus pyramidum floweri* (37/-; mean intragroup K2P distance = 0.004, Table 1), the other including the specimens attributed to the three other subspecies of *Gerbillus pyramidum* (i.e. *Gerbillus pyramidum pyramidum*, *Gerbillus pyramidum gedeedus* and *Gerbillus pyramidum elbaensis*; 55/0.98; mean intragroup K2P distance = 0.004, Table 1).

**Table 1.** K2P genetic distances between and within (in italic) taxa based on *cyt b* sequences.

	*Gerbillus pyramidum floweri* + *Gerbillus perpallidus*	*Gerbillus pyramidum*	*Gerbillus gerbillus*	*Gerbillus amoenus*	*Gerbillus nanus*	*Gerbillus andersoni*
*Gerbillus pyramidum floweri* + *Gerbillus perpallidus*	*0.004*					
*Gerbillus pyramidum*	0.017	*0.004*				
*Gerbillus gerbillus*	0.105	0.109	*0.013*			
*Gerbillus amoenus*	0.135	0.139	0.146	*0.011*		
*Gerbillus nanus*	0.112	0.116	0.138	0.037	*0.025*	
*Gerbillus andersoni*	0.113	0.114	0.126	0.103	0.106	*0.006*

## Discussion

We removed 7 individuals from the analyses, for which minor differences between sequences obtained from the amplified DNA coming from the two different concentrations of extracted DNA were observed. This observation strongly suggests the presence of nuclear copies of the target sequence, as is sometimes recorded in gerbilline rodents (Dobigny 2002, Ndiaye 2013).

Overall, the labelling of the museum samples was remarkably in line with current taxonomy, and all corresponding specimens appear coherently placed in the phylogenetic tree produced (but see below for further details). This validates *a posteriori* the “mini-barcode” method used. Indeed, the sequences obtained, although short (239bp) made it possible to obtain generally robust reconstructions of the phylogenetic relationships between the study specimens, thus confirming the findings of Hajibabaei et al. (2006) and Galan et al. (2012) on the usefulness of small fragments in molecular taxonomy. This also means that the *ad hoc* primers designed for this particular experiment are well-adapted for a molecular barcoding approach based on potentially degraded DNA in the study group.

Museum specimens of *Gerbillus gerbillus* and *Gerbillus andersoni* from Egypt clustered unambiguously with “fresh” specimens of the same species from other origins. As for the distinction between Asian *Gerbillus nanus* and African *Gerbillus amoenus*, even if supported here by non-optimal BP values, it confirms the findings of Ndiaye et al. (2013) and the hypothesis of two vicariant sibling species that could have differentiated in allopatry on both sides of the Red Sea. The museum specimens used here were labelled as *Gerbillus amoenus* following Osborn and Helmy (1980) who treated them (= *Dipodillus amoenus*) as a distinct species from *Gerbillus nanus* (= *Dipodillus nanus*). This taxonomy was not followed by many subsequent studies (see details in Ndiaye et al. 2013), but it now unambiguously appears that these two taxa have to be considered as distinct species. The inclusion of Egyptian specimens in the present study (clearly classified as *Gerbillus amoenus*) and the presence among the *Gerbillus nanus* sample of one reference specimen from Israel reinforce this conclusion, and confirmed that the Red Sea probably represents the geographical limit separating these two vicariant species.

**Figure 2.**
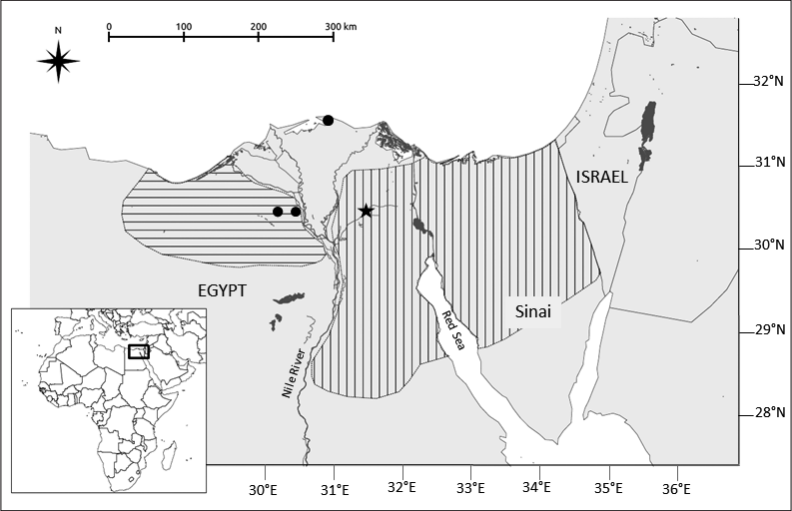
Reevaluated *Gerbillus floweri* distribution area based on the results presented here (see text; horizontal lines: *Gerbillus perpallidus*; vertical lines: *Gerbillus floweri* distributions according to the IUCN Red List of Threatened Species. Version 2015.2. www.iucnredlist.org). Black circles and black star: specimens of *Gerbillus perpallidus* and *Gerbillus pyramidum floweri*, respectively, used in the present study.

The series of museum specimens of large-sized and hairy-footed gerbils referred to as *Gerbillus perpallidus* and *Gerbillus pyramidum* ssp. were distributed into two moderately well supported genetic clades: the first one includes all *Gerbillus pyramidum floweri* and *Gerbillus perpallidus* samples, together with a reference specimen of *Gerbillus perpallidus* (namely Gperp1, underlined in Fig. 1 and Suppl. material 1). The sequences of these 10 specimens show a very high degree of similarity. *Gerbillus perpallidus*, described by Setzer (1958) in Egypt, is currently considered as endemic to Egypt, where it is distributed in a relatively small area west of the Nile delta (Happold 2013). It is listed as a valid species by most recent authors (Osborn and Helmy 1980, Lay 1983, Pavlinov et al. 1990; Musser and Carleton 2005) whereas Cockrum (1977) and Petter (1975) considered it as synonymous with *Gerbillus latastei* and *Gerbillus pyramidum*, respectively. Osborn and Helmy (1980) considered *Gerbillus floweri* (Thomas, 1919) as a subspecies of *Gerbillus pyramidum*, but it is generally listed as a valid species following the review of Lay (1983). It also has a relatively small distribution range in Northern Egypt, east of the Nile delta and in most of the Sinai Peninsula (Happold 2013). Interestingly, Osborn and Helmy (1980: 114) insisted on the morphological similarity between the two taxa, both based on body and skull characteristics: “*Gerbillus pyramidum floweri* and *Gerbillus perpallidus* are strikingly similar in color, bulla shape and, in some individuals, posterior margin of nasals”. Our results confirm these observations and suggest that these two taxa may represent a single species, the name of which should be *Gerbillus floweri*, according to his first description. This species would be characterized by a karyotype with a diploid number of chromosomes of 2n = 40, and an autosomal fundamental number NFa = 76, as described in Lay et al. (1975) and Aniskin et al. (2006) under *Gerbillus perpallidus*. The distribution of this species would then encompass both sides of the Nile delta in Northern Egypt, and extend through the whole of Northern Sinai (Fig. 2). Its sister species would be *Gerbillus pyramidum*, which confirms what was found in other recent studies. The genetic distance between these taxa, as shown by this study, appears to be very small (K2P = 0.017). It was larger in two other studies based on complete cyt*b* sequences of samples of different individuals of *Gerbillus pyramidum* and *Gerbillus perpallidus* only (K2P distance = 0.029 and 0.031, respectively, in Ndiaye et al. 2012; in review).

In addition to *Gerbillus pyramidum floweri*, Osborn and Helmy (1980) recognized three other subspecies in Egypt, namely *Gerbillus pyramidum pyramidum*, *Gerbillus pyramidum elbaensis* and *Gerbillus pyramidum gedeedus*. The museum samples that refer to these three subspecies cluster together in our analyses (with two reference samples of *Gerbillus pyramidum*), but they were intermixed in this clade, suggesting that these subspecies do not represent genetic lineages with independent evolutionary histories, at least based on partial sequences of cyt*b.* Their current allopatric distributions may be of relatively recent origin, following the last episode of drying of the Sahara, between 6,000 and 4,000 years ago (Holmes 2008). The geographic and / or adaptive morphological differentiation that has resulted from this separation may thus also be of recent origin, and the cyt*b* fragments sequenced here may not reflect this differentiation yet.

In conclusion, we show here that molecular analysis of historic museum samples of the genus *Gerbillus*, up to more than 100 years after their collection, may give useful information, and address testable hypotheses, about the systematics of the genus. This could aid in the completion of the taxonomic understanding of this complex and speciose genus, which is well represented in museum collections worldwide. The new primers specifically designed here, may prove useful for this purpose.

## Supplementary materials

Supplementary material 1 List of study specimens with identification numbers, geographic origin, collector name and collection date Data type: Information of study specimens and cytb sequences This dataset is made available under the Open Database License (http://opendatacommons.org/licenses/odbl/1.0/). The Open Database License (ODbL) is a license agreement intended to allow users to freely share, modify, and use this Dataset while maintaining this same freedom for others, provided that the original source and author(s) are credited.Arame Ndiaye, Caroline Tatard, William Stanley, Laurent Granjon
